# Transcriptome Analysis Unveils Molecular Mechanisms of Acaricide Resistance in Two-Spotted Spider Mite Populations on Hops

**DOI:** 10.3390/ijms252413298

**Published:** 2024-12-11

**Authors:** Sonu Koirala B K, Gaurab Bhattarai, Adekunle W. Adesanya, Timothy W. Moural, Laura C. Lavine, Douglas B. Walsh, Fang Zhu

**Affiliations:** 1 Department of Entomology, Pennsylvania State University, University Park, PA 16802, USA; svk6273@psu.edu (S.K.B.K.); twm78@psu.edu (T.W.M.); 2 Institute of Plant Breeding, Genetics & Genomics, University of Georgia, Athens, GA 30602, USA; gbhattarai@uga.edu; 3 Department of Entomology, Washington State University, Pullman, WA 99164, USAlavine@wsu.edu (L.C.L.); dwalsh@wsu.edu (D.B.W.); 4 Irrigated Agriculture Research and Extension Center, Washington State University, Prosser, WA 99350, USA; 5 Huck Institutes of the Life Sciences, Pennsylvania State University, University Park, PA 16802, USA

**Keywords:** acaricide resistance, two-spotted spider mite, RNA sequencing, metabolic detoxification

## Abstract

Broad-spectrum crop protection technologies, such as abamectin and bifenthrin, are globally relied upon to curb the existential threats from economic crop pests such as the generalist herbivore *Tetranychus urticae* Koch (TSSM). However, the rising cost of discovering and registering new acaricides, particularly for specialty crops, along with the increasing risk of pesticide resistance development, underscores the urgent need to preserve the efficacy of currently registered acaricides. This study examined the overall genetic mechanism underlying adaptation to abamectin and bifenthrin in *T. urticae* populations from commercial hop fields in the Pacific Northwestern region of the USA. A transcriptomic study was conducted using four populations (susceptible, abamectin-resistant, and two bifenthrin-resistant populations). Differential gene expression analysis revealed a notable disparity, with significantly more downregulated genes than upregulated genes in both resistant populations. Gene ontology enrichment analysis revealed a striking consistency among all three resistant populations, with downregulated genes predominately associated with chitin metabolism. In contrast, upregulated genes in the resistant populations were linked to biological processes, such as peptidase activity and oxidoreductase activity. Proteolytic activity by peptidase enzymes in abamectin- and bifenthrin-resistant TSSM populations may suggest their involvement in acaricide metabolism. These findings provide valuable insights into the molecular mechanisms underlying acaricide resistance in the TSSM. This knowledge can be utilized to develop innovative pesticides and molecular diagnostic tools for effectively monitoring and managing resistant TSSM populations.

## 1. Introduction

Hop (*Humulus lupulus*) is an economically important crop in the Pacific Northwest (PNW) of the United States, mostly recognized for its role in imparting flavor, fragrance, and preservative attributes to the brewing of beer [1,2]. The primary hop-producing states in the U.S. are Washington, Oregon, and Idaho. Among them, Yakima Valley in the state of Washington holds significant importance as one of the leading hop-growing regions globally [3]. The two-spotted spider mite (TSSM), *Tetranychus urticae* Koch, is the primary arthropod pest on hops, particularly under the hot and dry conditions of the Pacific Northwest, where 99% of U.S. hops are produced. The TSSM is a cell content feeder, utilizing the stylets of its mouthparts to penetrate leaf and cone tissues. Through this process, it extracts and depletes cell contents, ultimately resulting in cell death [4]. Upon continuous infestation, the damage can lead to the entire leaf turning bronze and drying out, eventually diminishing plant vigor and impacting overall yield [5]. Hot and dry weather of the PNW region provides favorable conditions for mite growth and reproduction [6]. As one of the most phytophagous herbivores, the TSSM feeds on over 1100 plant species [7,8]. Phytophagous herbivores have developed various strategies to adapt to host plants that contain a wide range of allelochemicals. These adaptations involve different mechanisms, facilitating the ability of herbivores to tolerate various toxins present in plants, highlighting the intricate coevolutionary relationship between herbivores and plants [9,10]. Moreover, such adaptive mechanisms may also contribute to the development of resistance to pesticides, suggesting a possible link between host plant adaptation and pesticide resistance in arthropods [11]. So far, the TSSM has evolved resistance to over 96 unique insecticides/acaricides, ranking it the most resistant of arthropod pest species (Arthropods resistant to Pesticides Database (ARPD), accessed on 23 September 2024).

Various TSSM management practices have been utilized to improve control effectiveness and support Integrated Pest Management (IPM) practices in hops. Some recommended IPM practices for TSSM management in hops include adequate nitrogen fertilization, minimizing the dispersal of dust around the field, growing cover crops that provide habitats for natural enemies such as predatory mites, and the application of selective miticides [5]. The use of predatory phytoseiid mites for biological control has been implemented as a promising approach [12,13,14]. However, acaricide spray remains the primary method for TSSM management due to its effectiveness within short application periods [15]. Grower records indicate that as many as nine pesticides are utilized throughout each hop-growing season annually [1,16]. Frequent acaricide use has driven the development of broad cross-resistance, prompting the search for new acaricides and leading to increased use of higher doses [14,17]. The rapid development of acaricide resistance in the TSSM is likely facilitated by its extremely short life span, small body plan, high fecundity, haplo-diploid sex determination, and fast growth and maturation [18,19]. The combination of multiple resistance mechanisms can result in a high degree of pesticide resistance [20]. 

Like other arthropods, the TSSM employs several mechanisms to adapt to their chemical environment, including enhanced metabolic detoxification, target site insensitivity, behavioral avoidance, and decreased cuticular penetration [2,21,22,23,24]. Among these mechanisms, enhanced metabolic detoxification and target site insensitivity have been mostly reported in the TSSM [25,26,27]. Enhanced metabolic detoxification involves reducing the concentration of parent or intermediate toxic compounds before they reach their target sites, by strengthening detoxification processes. Conversely, target site insensitivity involves target sites altered through mutations, thereby diminishing the binding affinity of pesticides to target proteins [28]. Studies have shown that when arthropods were exposed to pesticides or harmful chemicals, their bodies responded by upregulating the activity of detoxification genes. The *cis*- or *trans*-regulation of gene expression or mutations in the catalytic sites of detoxification proteins [29,30,31,32] help the arthropods break down and eliminate these toxic substances [33,34]. Additionally, the upregulation of detoxification genes can be constitutive or inducible, which might play a role in cross-resistance development [35,36]. Detoxification enzymes, including cytochrome P450 monooxygenases (CYPs), glutathione S-transferases (GSTs), carboxylesterases (CCEs), ATP-binding cassette (ABC) transporters, and Uridine diphosphate (UDP)-glycosyltransferases (UGTs), are known to be associated with acaricide/insecticide resistance in various insect and mite species [8,18,21,37,38,39,40,41]. Target site insensitivity results from alterations in the target protein, typically caused by amino acid substitutions. Such structural modification can lead to resistance to or, in certain instances, increased sensitivity towards toxic chemicals [35]. Resistance mutations in the voltage-gated sodium channel to pyrethroids, acetylcholinesterase genes to organophosphates, GABA-gated chloride channel to organochlorines and phenylpyrazoles, cytochrome b (Cytb) to bifenazate, and glutamate-gated chloride channels (GluCls) to abamectin have been identified in TSSM populations associated with acaricide resistance [18,28,42,43,44,45,46]. Both the increased expression of detoxification genes and mutations that alter gene expression can be used as molecular tools for identifying and tracking acaricide resistance in the field [1,16,47]. 

Acaricides used in TSSM management in the PNW have various modes of action. Among them, abamectin and bifenthrin are the two most applied acaricides in hopyards [1,47]. Abamectin is a neurotoxic pesticide belonging to IRAC Group 6 of glutamate-gated chloride channel (GluCl) allosteric modulators [48]. Numerous studies have found abamectin resistance associated with mutations in GluCls and increased metabolic detoxification in the TSSM [27,46,49,50]. However, in hopyards, the enhanced expression of metabolic detoxification genes is associated with abamectin-resistant TSSM populations with no evidence of mutations in the glutamate-gated chloride channel protein [1,16]. Bifenthrin is a pyrethroid pesticide that acts as a modulator of voltage-gated sodium channels and is classified as a Group 3 insecticide by the Insecticide Resistance Action Committee (IRAC) [48]. Bifenthrin resistance in TSSM populations from hops has been mainly linked with amino acid substitutions in voltage-gated sodium channels. Moreover, higher expressions of metabolic genes may also play a role in contributing to this resistance [1,16,51]. However, the overall molecular mechanisms underlying abamectin and bifenthrin resistance in TSSM populations from hop fields remain largely unknown.

In this study, we collected field samples of acaricide-resistant TSSM populations from the hop-growing region of Yakima Valley in Washington State, reared them in the lab and selected them for resistance against two commercial acaricides (abamectin and bifenthrin) over 40–80 generations. Total RNA was extracted from both acaricide-resistant and -susceptible TSSM populations, and comparative transcriptomic and variant analyses were used to identify the molecular mechanisms of resistance against two acaricides. We investigated members of various detoxification gene classes, their expression patterns, and the occurrence of functionally important mutations that may confer acaricide resistance in TSSM populations on hops. The results of this study enhance our understanding of the molecular mechanisms of acaricide resistance in TSSMs, which will facilitate the development of molecular diagnostic tools for acaricide resistance and effective management of TSSMs in hop fields.

## 2. Results

### 2.1. Development of Acaricide-Resistant TSSM Populations

All three acaricide-resistant and -susceptible TSSM populations were subjected to abamectin and bifenthrin treatment for bioassays as described before [52] (Table S1). Under abamectin treatment, bifenthrin-resistant TSSM populations at 1X and 100X doses showed LC50 of 8.6 and 13.2 ppm a.i. These LC50s are 10.8 and 16.5 times higher than the LC50 of the susceptible population and 18.2 and 11.9 times lower than the LC50 of the abamectin-resistant TSSM population, respectively. Under bifenthrin treatment, BIF_1X and BIF_100X populations showed LC50s of 362.8 and 1340.0, respectively, which are 20.1 and 74.4 times higher than the susceptible LC50 and 6.0 and 22.3 times higher than the abamectin-resistant population (ABA_1X), respectively. The bioassay results indicate that although abamectin- and bifenthrin-resistant populations clearly showed higher levels of resistant response when exposed to the corresponding acaricides, their higher cross-resistance ratio against two acaricides indicates a possible similarity in the molecular mechanism in conferring cross-resistance. In that regard, this experimental design is valid and presents the basis for identifying the molecular mechanisms of acaricide resistance in TSSMs.

### 2.2. RNA Sequencing and Alignment

RNA sequencing yielded approximately 298.8 million paired reads from 12 samples of acaricide-resistant and -susceptible TSSM populations (three biological replicates per sample). This resulted in an average of approximately 24.9 million read pairs per sample, ranging from 18,504,879 to 26,987,684 read pairs across 12 samples. On average, 0.034% of the raw reads were filtered by TrimGalore for minimum read quality and length. On average, 84.09% of the filtered reads aligned to the *T. urticae* reference genome (Table 1 and Table S2) [8].

**Figure 1:**
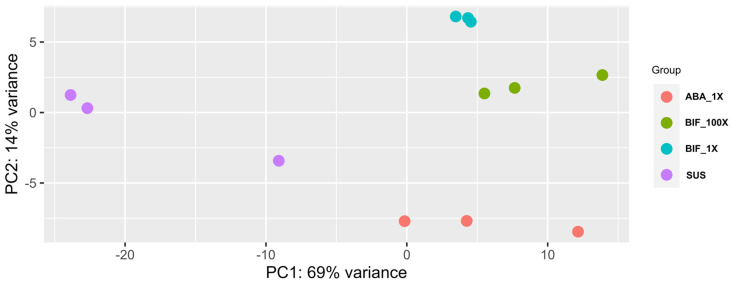
Principal component analysis (PCA) of variance-stabilization-transformed (vst) normalized gene expression values in all three biological replicates of acaricide-resistant and -susceptible TSSM populations. The first (PC1) and second (PC2) principal components explain 69% and 14% of the total variance observed for gene expression, respectively. Each colored dot represents a biological replicate. SUS: susceptible; ABA_1X: abamectin-resistant; BIF_1X and BIF_100X: bifenthrin-resistant.

There was a clear distinction between susceptible and acaricide-resistant TSSM populations for their gene expression pattern (Figure 1). A principal component analysis of normalized gene expression showed the clear clustering of susceptible and acaricide-resistant samples along the first principal components, explaining 69% of the total variance in gene expression among the samples. This indicates that gene expression in acaricide-resistant TSSM strains differs largely from that of the susceptible strains. Biological replicates of abamectin- and bifenthrin-resistant samples separated along the second principal component, which explains 14% of the total variance, indicating there may exist certain differences in the transcriptomic response of TSSM populations’ resistance to two different acaricides. Certain similarities may exist in the molecular mechanisms of TSSM resistance against abamectin and bifenthrin acaricides. In Figure 1, the PCA shows greater diversity in the susceptible strains compared to the resistant strains. Extended acaricide selection pressure on resistant populations often leads to reduced genetic diversity as only individuals with specific resistance traits are favored, which can result in less variation in the resistant strains.

### 2.3. Differential Gene Expression in Acaricide-Resistant TSSM Populations

**Figure 2:**
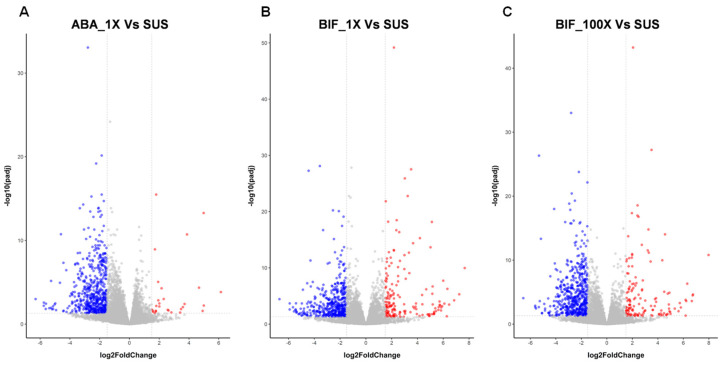
Volcano plots showing differentially expressed genes (|log_2_fold change| ≥ 1.5) in (**A**) abamectin- and (**B**,**C**) bifenthrin-resistant two-spotted spider mite (TSSM) populations. Orange and blue dots represent upregulated (log_2_fold change ≥ 1.5) and downregulated (log_2_fold change ≤ −1.5) genes (FDR-adjusted *p*-value < 0.05), respectively. SUS: susceptible; ABA_1X: abamectin-resistant; BIF_1X and BIF_100X: bifenthrin-resistant.

In total, 597, 635, and 679 genes were differentially expressed in ABA_1X, BIF_1X, and BIF_100X TSSM populations, respectively (Table 2, Figure 2). However, the number of upregulated genes in the abamectin-resistant population was notably less than those in bifenthrin-resistant TSSM populations. Only 28 genes were upregulated (log_2_fold ≥ 1.5) in ABA_1X, whereas 166 and 139 genes were upregulated in BIF_1X and BIF_100X, respectively. The differences in the number of upregulated genes between abamectin- and bifenthrin-resistant TSSM populations may indicate that there are differences in the molecular mechanisms of resistance against these two different acaricides. 

**Figure 3:**
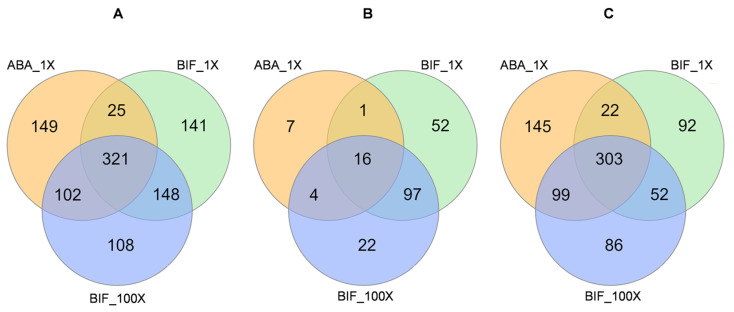
Venn diagrams showing (**A**) all differentially expressed (both up- and downregulated), (**B**) only upregulated, and (**C**) only downregulated common and unique genes among three acaricide-resistant two-spotted spider mite (TSSM) populations: ABA_1X, BIF_1X, and BIF_100X, respectively. ABA_1X: abamectin-resistant; BIF_1X and BIF_100X: bifenthrin-resistant.

An analysis of shared and unique differentially expressed genes (DEGs) showed that 398 genes were expressed in only one population (149 in ABA_1X, 141 in BIF_1X, and 108 in BIF_100X, respectively), 275 genes were found common in two resistant populations (25 in ABA_1X and BIF_1X, 102 in ABA_1X and BIF_100X, and 148 in BIF_1X and BIF_100X), and 321 genes were common in all three acaricide-resistant populations (Figure 3A). Sub-setting DEGs into up- and downregulated gene categories showed that 81 genes were upregulated in only one population (7 in ABA_1X, 52 in BIF_1X, and 22 in BIF_100X), 102 genes were found commonly upregulated in two resistant populations (1 in ABA_1X and BIF_1X, 4 in ABA_1X and BIF_100X, and 97 in BIF_1X and BIF_100X), and 16 genes were upregulated commonly in all three acaricide-resistant TSSM populations (Figure 3B). Among 799 unique downregulated genes in all acaricide-resistant TSSM populations, 323 genes were downregulated in only one population (145 in ABA_1X, 92 in BIF_1X, and 22 in BIF_100X), 173 genes were commonly downregulated in two resistant populations (22 in ABA_1X and BIF_1X, 99 in ABA_1X and BIF_100X, and 52 in BIF_1X and BIF_100X), and 303 downregulated genes were common in all three resistant populations (Figure 3C). The sixteen genes commonly upregulated in all three acaricide-resistant populations mostly belong to the protease and protease inhibitor class, such as cathepsin, legumain, and serpin 3 inhibitory serine protease inhibitors (Table S3). Upregulated genes shared only between BIF_1X and BIF_100X consisted of genes belonging to several classical detoxification gene families, including glutathione S-transferases (GSTs), cytochrome P450s (CYPs), carboxyl/cholineesterases (CCEs), and UDP-glycosyltransferases (UGTs) (Table S3). In addition, several other classes of genes, including proteases, were commonly upregulated in two bifenthrin-resistant TSSM populations.

### 2.4. Gene Ontological Pattern

**Figure 4:**
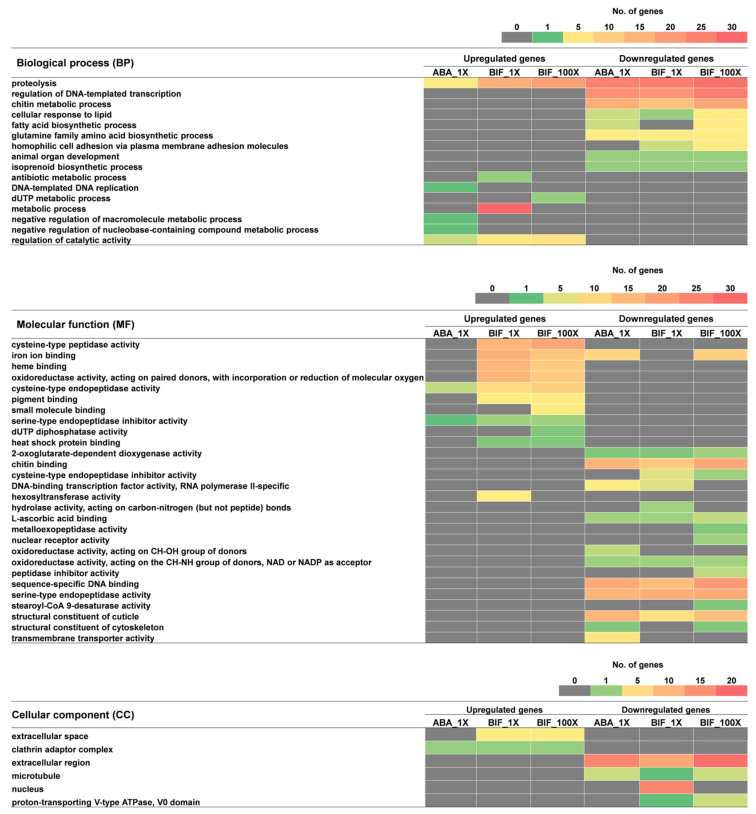
The ontological relationship of up- and downregulated genes in three acaricide-resistant populations. The gene ontology is presented as biological processes (BPs) (**top**), molecular functions (MFs) (**middle**), and cellular components (**bottom**), and the color scales represent the number of differentially expressed genes in the corresponding gene ontology. Up- and downregulated genes were selected based on the following criteria: |log_2_fold change| > 1.5 and Benjamini–Hochberg (BH)-adjusted *p*-values < 0.05.

A gene ontology enrichment analysis of upregulated genes showed that the biological processes (BPs) of proteolysis and regulation of catalytic activity were significantly enriched in all three acaricide-resistant TSSM populations (Figure 4). In contrast, processes such as DNA-templated DNA replication, negative regulation of macromolecule metabolic processes, and negative regulation of nucleobase-containing compound metabolic processes were unique to ABA_1X. Similarly, metabolism-related processes such as antibiotic metabolic processes, dUTP metabolic processes, and metabolic processes were significantly enriched and unique to bifenthrin-resistant populations. In comparison, except for proteolysis, genes related to significantly enriched biological processes, such as the regulation of DNA-templated transcription, chitin metabolic processes, cellular responses to lipid, fatty acid biosynthetic processes, glutamine family amino acid biosynthetic processes, homophilic cell adhesion via plasma membrane adhesion molecules, animal organ development, and isoprenoid biosynthetic processes were downregulated in acaricide-resistant TSSM populations (Figure 4). Among these processes, the fatty acid biosynthetic processes and homophilic cell adhesion via plasma membrane adhesion molecules processes were common in all three acaricide-resistant populations, indicating the downregulation of genes involved in similar biological processes in both abamectin and bifenthrin resistance. 

Significantly enriched molecular functions (MFs) of cystine-type endopeptidase activity and serine-type endopeptidase inhibitor activity were common in all three acaricide-resistant populations, and genes related to these functions were upregulated. Also, these are the only molecular functions significantly enriched in ABA_1X. In contrast, genes related to molecular functions, such as cystine-type peptidase activity, iron ion binding, heme binding, oxidoreductase activity (acting on paired donors, with incorporation or reduction of molecular oxygen), pigment binding, and heat shock protein binding were upregulated in both BIF_1X and BIF_100X populations. Additionally, the molecular functions of small-molecule binding and dUTP diphosphatase activity were unique to BIF_100X, while hexosyltransferase activity was unique to BIF_1X. Genes related to molecular functions shared between all three acaricide-resistant TSSM populations, including 2-oxoglutarate-dependent dioxygenase activity, chitin binding, L-ascorbic acid binding, oxidoreductase activity (acting on the CH-NH group of donors, NAD or NADP as an acceptor), sequence-specific DNA binding, serine-type endopeptidase activity, and the structural constituents of cuticles were downregulated. Upregulated genes in all acaricide-resistant populations were found to be a part of the clathrin adaptor complex. Genes upregulated only in two bifenthrin-resistant populations showed their potential function in the extracellular space. The enrichment of these cellular components indicates the role of endocytosis and cellular signaling in acaricide resistance in the TSSM.

### 2.5. Expression of Classical Detoxification Genes

**Figure 5:**
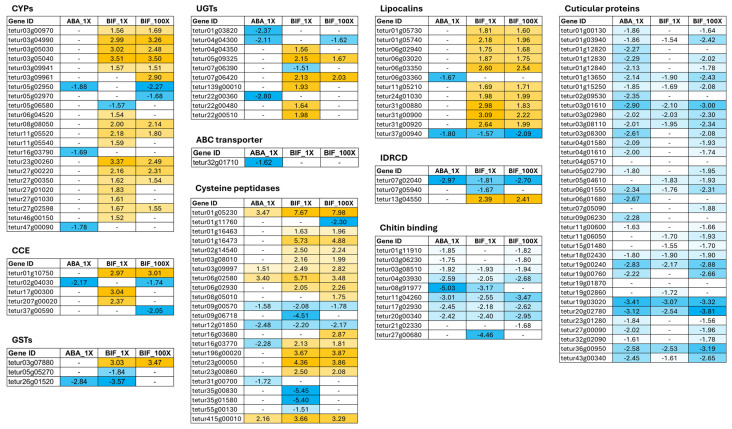
log_2_fold change in genes belonging to various detoxification gene classes in abamectin (ABA_1X)- and bifenthrin (BIF_1X and BIF_100X)-resistant TSSM populations. The value “-” indicates that the gene does not meet the statistical criteria to be called differentially expressed in this study (|log_2_fold| ≥ 1.5 and Benjamin–Hochberg-adjusted contrast *p*-value ≤ 0.05). The color intensity in each gene category indicates the level of gene expression, with darker shades representing more |log_2_fold| changes. Dark blue indicates greater downregulation, while dark orange denotes higher upregulation. ABA_1X: abamectin-resistant; BIF_1X and BIF_100X: bifenthrin-resistant.

Several detoxification enzymes associated with TSSM resistance to pesticides have been discovered [8]. However, no upregulation of genes encoding detoxification enzymes was detected in ABA_1X in this study, indicating the potential involvement of distinct pathways in abamectin resistance development in TSSMs (Figure 2A). In BIF_1X, 16 out of a total of 86 P450s genes were upregulated, including cytochrome P450 fragment (tetur03g05040) and cytochrome P450 probable pseudogene (tetur27g00220) (Figure 5). For BIF_100X, 12 CYPs showed upregulation relative to the susceptible population, with the majority showing similar expression levels to those observed in BIF_1X. In the TSSM, the carboxyl/cholinesterase (CCE) gene family comprises 71 genes [8]. Three CCE genes (tetur17g00300, tetur01g10750, and tetur207g00020) were upregulated in BIF_1X. However, only one CCE (tetur01g10750) was differentially expressed (upregulated) in BIF_100X, with its expression level similar to that observed in BIF_1X. A single GST (tetur03g07880) exhibited upregulation in BIF_1X and BIF_100X populations, with log_2_fold changes of 3.04 and 3.47, respectively (Figure 5). In BIF_1X, a total of six UGTs (tetur05g09325, tetur22g00510, tetur139g00010, tetur22g00480, tetur04g04350, and tetur07g06420) showed upregulation, while in BIF_100X, only two UGTs (tetur05g09325 and tetur07g06420) were upregulated. No upregulation of ABC transporter genes was observed in abamectin- or bifenthrin-resistant TSSM populations (Figure 5).

### 2.6. Expression of Peptidases 

Peptidase genes were among the upregulated genes common across three acaricide-resistant TSSM populations. Peptidase-encoding genes belonging to the cysteine, serine, and aspartic groups have been identified in the TSSM genome [53]. Our findings indicate that most upregulated peptidases were cysteine-based, particularly C1A papain and C13 legumain genes. Three genes (tetur01g05230, tetur03g09997, and tetur06g02580) classified as C1A papain, and one C13 legumain gene (tetur415g00010) was upregulated in both abamectin- and bifenthrin-resistant populations (Figure 5). The log_2_fold change for C1A papain and C13 legumain was greater in BIF_1X and BIF_100X compared to ABA_1X. A total of eleven C1A papain genes (tetur01g16463, tetur01g16473, tetur02g14540, tetur03g08010, tetur03g09997, tetur06g02580, tetur06g02930, tetur16g03770, tetur196g00020, tetur23g00050, and tetur23g00860) were upregulated in BIF_1X and BIF_100X, with no differential expression observed in ABA_1X (Figure 5). Two genes (tetur08g05010 and tetur16g03680) were upregulated exclusively in BIF_100X, with no differential expression in BIF_1X or ABA_1X. 

### 2.7. Expression of Cuticular Proteins, Chitin-Binding Proteins, and Chitinases

Chitin-binding and cuticular proteins are crucial constituents of cuticles, while chitinases play a role in degrading the old cuticle [54,55]. The cuticle serves multiple functions, such as shaping the body, preventing water loss, and providing a protective outer barrier against environmental stress [56]. Our results indicate that there was a decrease in the expression of cuticular proteins and chitin-binding proteins in both abamectin- and bifenthrin-resistant populations compared to the susceptible population (Figure 5).

### 2.8. Expression of Laterally Transferred Genes

Intradiol ring-cleavage dioxygenases (IDRCDs) and genes involved in carotenoid biosynthesis have been identified and considered to have been acquired through lateral gene transfer from bacteria and fungi [8,11]. IDRCDs are involved in the metabolization of aromatic compounds by cleaving the aromatic ring between two adjacent hydroxyl groups by utilizing a non-heme iron cofactor in the active site. Seventeen IDRCD genes were identified in the *T. urticae* genome. IDRCDs are known for their involvement in metabolizing aromatic compounds found in plant allelochemicals, while carotenoids initiate diapause induction [8,57]. In the abamectin-resistant population, one IDRCD gene, tetur07g02040, was downregulated. However, in the BIF_1X and BIF_100X populations, tetur13g04550 was upregulated. Two IDRCD genes, tetur07g02040 and tetur07g05940, were downregulated in BIF_1X and BIF_100X populations. Lipocalin is an another group of laterally transferred gene upregulated in the resistance population [58]. A total of 12 lipocalin-encoding genes were differentially expressed in our study: 10 were upregulated and 1 was downregulated in BIF_1X and BIF_100X (Table S3; Figure 5). No lipocalin-encoding genes were upregulated; instead, two genes, tetur06g03360 and terur37g00940, were downregulated with a log_2_fold of −1.67 and −1.80, respectively, in ABA_1X (Figure 5).

### 2.9. Variant Identification and Annotation

**Figure 6:**
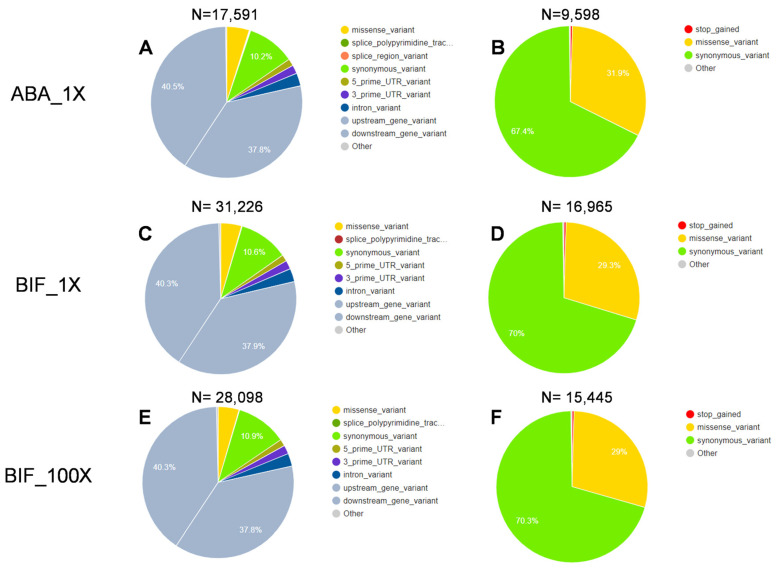
Percentage of (**A**,**C**,**E**) single-nucleotide polymorphisms (SNPs) with all consequences and (**B**,**D**,**F**) consequences in coding regions in the ABA_1X, BIF_1X, and BIF_100X TSSM populations, respectively. SNPs were identified in resistant populations in reference to the homozygous genotype of the susceptible TSSM population. N: number of variants. ABA_1X: abamectin-resistant; BIF_1X and BIF_100X: bifenthrin-resistant.

In total, 17,591, 31,226, and 28,098 single-nucleotide polymorphisms (SNPs) were identified in the ABA_1X, BIF_1X, and BIF_100X TSSM populations, respectively, when compared with the homozygous genotype of SNPs in the SUS population (Figure 6). Among them, 9598, 16,965, and 15,445 SNPs were identified within coding regions in the ABA_1X, BIF_1X, and BIF_100X populations, respectively. Of these, 31.9% (3062), 29.3% (4971), and 29% (4478) had missense-type mutations in the coding region in three acaricide-resistant TSSM populations, respectively (Figure 6). An analysis of unique and shared missense mutations in three acaricide-resistant populations showed that 2760 missense mutations were identified in only one population (846 in ABA_1X, 1208 in BIF_1X, and 706 in BIF_100X), 2466 missense mutations were common in two acaricide-resistant populations (308 in ABA_1X and BIF_1X, 317 in ABA_1X and BIF_100X, and 1841 in BIF_1X and BIF_100X), and 1548 missense mutations were common in all three acaricide-resistant populations (Figure S1). We also performed a gene ontology enrichment analysis of genes with missense mutations in all three acaricide-resistant populations (Figure S2). Two biological processes—metabolic processes and the regulation of gene expression—were among the highest enriched biological processes and common in all three acaricide-resistant populations.

## 3. Discussion

Enhanced metabolic detoxification is one of the major mechanisms involved in pesticide resistance in various insect and mite species, including the TSSM [20,47,59,60,61,62,63,64,65,66]. This mechanism manifests through upregulated gene expression, gene amplification or mutations in the genes coding detoxification enzymes, leading to higher levels of enzyme activity, capable of degrading or sequestering pesticides more effectively [30,38,67,68,69,70]. The known detoxification enzymes associated with the metabolism of xenobiotics are Phase I, II, and III enzymes, including CYPs, GSTs, CCEs, ABC transporters, and UGTs [39,71,72]. Our study showed the upregulation of multiple CYPs, CCEs, GSTs, and UGTs in the BIF_1X and BIF_100X populations compared to the susceptible one, suggesting that enhanced metabolic detoxification may play a critical role in bifenthrin resistance in hop TSSM populations. However, most of the classical detoxification enzymes in ABA_1X were downregulated, indicating enhanced metabolic detoxification may not be an important mechanism contributing to resistance against this class of acaricide in TSSM populations on hops. CYPs are important metabolic detoxification enzymes that are crucial for detoxification pesticides and plant allelochemicals, leading to the development of pesticide resistance and facilitating the adaptation of herbivores to their host plants [73]. Of the 86 CYP genes identified in the TSSM genome [8], 22 showed statistical evidence of differential gene expression in this study. Among them, one gene in the CYP3 clan (tetur11g05520), eight genes in the CYP2 clan (tetur03g00970, tetur03g04990, tetur03g05030, tetur03g09941, tetur08g08050, tetur23g00260, tetur27g00350, and tetur27g02598), and two uncharacterized CYP genes (tetur03g05040 and tetur27g00220) were upregulated in both the BIF_1X and BIF_100X populations (Table S3; Figure 5). Piraneo and others reported that *CYP385C4* (tetur11g05520) was highly upregulated in hop field-collected acaricide-resistant TSSM samples compared to the susceptible population, indicating its potential role in acaricide resistance [1]. The upregulation of *CYP392A11* (tetur03g00970) was also identified against two mitochondrial electron transport inhibitor (METI) class acaricides in cyenopyrafen- and fenpyroximate-resistant TSSM strains [74,75]. A functional analysis revealed its crucial role in metabolizing both cyenopyrafen and fenpyroximate [76]. In a follow-up study, the potential role of CYP392A11 in metabolizing another METI class acaricide, bifenazate, by the hydroxylation of the ring structure was reported [77]. In a transcriptomic investigation of TSSMs subjected to 20 generations of selection with bifenthrin on cowpea, the upregulation of *CYP392A11* and *CYP392D2* (tetur03g04990) was observed [78]. Moreover, *CYP392D2* was overexpressed in multiple acaricide-resistant TSSM populations on wild or greenhouse ornamentals and crop plants [77,79,80,81]. In our research, both *CYP392A11* and *CYP392D2* were significantly upregulated in bifenthrin-resistant populations compared to the susceptible population, whereas their expression levels were not affected by increased bifenthrin selection (1X – 100X). Another transcriptomic study showed that *CYP392A13v1* (tetur08g08050) and *CYP392A15* (tetur03g09941) were overexpressed in spiromesifen-, spirodiclofen-, and chlorfenapyr-selected TSSM populations collected from greenhouses [79,80] as well as a pyflubumide (another METI class acaricide) selected TSSM strains [44]. 

Besides CYPs, our study found the upregulation of CCEs and GSTs in the BIF_1X and BIF_100X populations. *TuCCE45* (tetur17g00300), *TuCCE04* (tetur01g10750), and *CCEincTu16* (tetur207g00020) were upregulated by a log_2_fold of 3.04, 2.97, and 2.37 in both the BIF_1X and BIF_100X populations (Table S3; Figure 5). The upregulation of *TuCCE04* has been found in cyenopyrafen-selected TSSMs, indicating its potential role in acaricide resistance [74]. In bifenthrin-selected TSSMs, *CCEincTu16* and *TuCCE45* were upregulated, which is consistent with our findings [78]. However, the functions of these CCEs associated with acaricide resistance have not been studied in TSSMs. One incomplete GST *TuGSTinc03* (tetur03g07880) was upregulated with a log_2_fold of 3.03 and 3.47 in BIF_1X and BIF_100X, respectively, indicating the potential role of this GST in bifenthrin resistance in hop TSSM populations. In addition, *TuGSTinc05* (tetur26g01520) was downregulated in both ABA_1X and BIF_1X, and *TuGSTm10* (tetur05g05270) was downregulated only in BIF_1X (Table S3; Figure 5). Of the 103 ATP-binding cassette (ABC) transporter encoding genes in the TSSM genome, *TuABCH-20* (tetur32g01710) was the only one that was downregulated in ABA_1X (Table S3; Figure 5). This suggests that ABC transporters may not be a major detoxification enzyme contributing to abamectin or bifenthrin resistance in hop TSSM populations.

UGTs are another type of Phase II detoxification enzymes that catalyze the conjugation reaction between UDP sugars and small hydrophobic molecules, generating more hydrophilic compounds [82]. A total of 80 UGTs have been identified in the TSSM genome [83]. In our research, 10 UGTs were differentially expressed, mostly in BIF_1X with a few in BIF_100X and ABA_1X (Figure 5). Among them, three UGTs (tetur01g03820, tetur04g04300, and tetur22g00360) were downregulated in ABA_1X, two UGTs (tetur05g09325 and tetur07g06420) were upregulated in BIF_1X and BIF_100X, and four UGTs (tetur04g04350, tetur139g00010, tetur22g00480, and tetur22g00510) showed significant upregulation only in BIF_1X (Table S3; Figure 5). A study has shown the upregulation of tetur139g00010 in field-collected TSSM populations with a history of spiromesifen application and selection [79]. When compared with acaricide-susceptible TSSMs, the same gene was found to be upregulated by 12.04- to 167.73-fold in field-collected TSSM samples with a history of exposure to abamectin, bifonazole, and clofentezine [84]. However, tetur139g00010 showed no difference in expression in the abamectin-resistant hop TSSM population in this study. The role of UGTs in acaricide resistance in hop TSSM populations requires further investigations. 

In this study, genes encoding cysteine peptidases, specifically C1A papain (identified as tetur03g09997, tetur01g05230, and tetur06g02580) and C13 legumain genes (tetur415g00010) exhibited increased expression in both abamectin- and bifenthrin-resistant populations. Through nano-LC-MS/MS analysis of the proteomic composition of TSSMs, the proteins encoded by tetur01g05230 and tetur06g02580 were detected in the TSSM saliva when the mites were adapted to different host plants, such as maize and tomato [85]. This suggests that these proteins may play a role in plant–mite interactions. C1A papain and C13 legumain are mostly known as digestive enzymes in mites, ticks, and insects [86,87,88,89]. Cysteine peptidase’s functions have been diversely described with their involvement in metamorphosis, ovarian maturation, and embryogenesis [90,91,92]. Regarding its physiological functions, cysteine peptidases were upregulated in *Bombyx mori* exposed to H_2_O_2_ and high temperature, resulting in premature larval–pupal transformation [93]. In our study, most of the C1A papain and C13 legumain were expressed at higher levels in the BIF_1X and BIF_100X populations compared to the susceptible population (Table S3; Figure 5). Exposure to organophosphates insecticide induced protease activities in the insecticide-resistant housefly *Musca domestica* [94] and silkworm [95]. It has been shown under in vitro conditions that some pesticides inhibit some types of proteases. However, given the vital roles of these enzymes, the increased expression of proteases may provide metabolic resistance against pesticides [95]. Increased protease activity enhances the abundance of free amino acids following dietary protein digestion, which might be used to synthesize other detoxification enzymes such as CYPs, GSTs, and esterases [95,96]. This also likely explains mites’ balance and energy trade-off from stress caused by acaricide exposure. The upregulation of various C1A papain and C13 legumain genes in response to different acaricide treatments, such as selection with abamectin and bifenthrin, underscores the potential role of these genes in contributing to abamectin and bifenthrin resistance in hop TSSM populations. 

In the TSSM genome, 58 lipocalin-encoding genes have been annotated [11]. Lipocalins are small extracellular proteins with a role in the binding and transportation of small hydrophobic molecules (such as retinol), coloration, olfaction, pheromone transport, and the enzymic synthesis of prostaglandins [97]. Lipocalins have been expressed in mites when exposed to pesticides and during host shifts, which play an important role in reducing stress created by pesticides and host shifts [11]. In this RNAseq analysis comparing susceptible and abamectin- and bifenthrin-resistant hop TSSM populations, two lipocalin genes (ApoD28 and ApoD29; tetur01g05730 and tetur01g05740) exhibited significantly higher expressions in bifenthrin-resistant populations than in the susceptible population (Figure 5). This upregulation was not limited to abamectin and bifenthrin resistance; the same genes were also highly expressed in TSSM populations resistant to other acaricides, specifically spirodiclofen and cyenopyrafen [74,98]. The consistent upregulation of these lipocalin genes across various resistant populations indicates a potential general mechanism of resistance. Lipocalins may contribute to the detoxification process or protection against oxidative stress induced by acaricides [11,99,100]. Their role in stress response suggests that these proteins could help mitigate the harmful effects of both pesticides and environmental changes, aiding in the survival and adaptation of resistant mite populations.

Out of 17 intradiol ring-cleavage dioxygenases (IDRCDs), 3 of them were differentially expressed in our study. IDRCDs belong to ring-cleavage dioxygenase enzyme families and are mostly known to cleave the aromatic ring between two adjacent hydroxyl groups [101,102]. Studies have highlighted the potential roles of IDRCDs in embryonic development, diapause induction, digestion, and the detoxification of aromatic compounds [103,104]. TuDOG11 (tetur13g04550) was the only common IDRCD upregulated in BIF_1X and BIF_100X with a log_2_fold of 2.39 and 2.41, respectively (Table S3; Figure 5). This observation, consistent with previous research showing the upregulation of this gene in various acaricide-resistant TSSM populations and in response to host plant adaptation [81,105], suggests that this gene may contribute to the development of acaricide resistance in hop TSSM populations.

Cuticle thickness affects mites’ tolerance to acaricides. Cuticular proteins have been shown to thicken the cuticle and reduce the penetration of toxic chemicals, thus inducing cuticle resistance [106]. Surprisingly, of 45 cuticle proteins annotated in the TSSM genome [8], most of them (36) were differentially expressed in our study (Figure 5). Both abamectin- and bifenthrin-resistant TSSMs had the largest number of DEGs for cuticular proteins; however, all of them were downregulated (Figure 5). Similarly, chitin-binding proteins were downregulated in both abamectin- and bifenthrin-resistant populations. In previous studies, such downregulation of cuticular proteins has also been observed in pesticide-resistant insects. Mevinphos-resistant *Plutella xylostella*, when compared with susceptible strains, showed that 12 out of 16 cuticular proteins were downregulated [107]. *Sitobion avenae* treated with imidacloprid and chlorpyrifos for 36 h showed the downregulation of 46 out of 50 and 40 out of 43 cuticular genes, respectively [108]. 

Trade-offs typically arise when there is insufficient allocation of resources required for reproduction, growth, and survival [109]. The most important tissues that need the most protection in response to damage need more resources [109,110]. According to resource allocation theory, organisms can distribute their resources according to various priorities. While doing so, the investment of resources decreases for other functions or traits [110,111,112]. The insect body is occupied mostly by the exoskeleton, and its production may be costly [109,113]. The downregulation of cuticular and chitin-binding protein-encoding genes in abamectin- and bifenthrin-resistant TSSM populations in this research, which are important constituents for making cuticles, might be due to fitness costs. Additionally, increased expression of lipocalin-encoding genes has been linked with the transport and deposition of surface cuticular lipids that are crucial for maintaining normal cuticle barrier function [114]. The upregulation of lipoprotein and downregulation of cuticle protein encoding genes observed in this study may indicate the balance between two processes without compromising the state of the normal cuticle barrier and thus may contribute to acaricide resistance in TSSMs. To further validate these findings and their implications, we plan to perform functional analysis studies in the future.

Another potential mechanism through which arthropods develop resistance to pesticides involves mutations in detoxification proteins [29,115]. When mutations occur in the genes encoding these proteins, the resulting amino acid changes can alter the structure and function of the enzyme [116]. In this transcriptomic study, SNP calling followed by GO enrichment analysis showed that a larger number of missense mutations occurred in genes that play roles in metabolic processes in both abamectin- and bifenthrin-resistant populations (Figure S2). The genes involved in metabolic processes were mostly classical detoxification enzymes including CYPs, CCEs, ABC transporters, UGTs, and GSTs. Mutations in any detoxification enzymes can either modify their catalytic activity, potentially altering the metabolism of pesticides, or have no functional impact [30,117,118]. More functional studies are required to elucidate the role of these missense mutations individually. 

In summary, we characterized abamectin resistance in the hop TSSM population by documenting the upregulation of several cysteine peptidases. Conversely, bifenthrin resistance may be associated with the upregulation of various genes, including genes coding for detoxification enzymes, peptidases, and lipocalins. In addition, the downregulation of cuticular proteins and chitin-binding proteins could also contribute to the development of abamectin and bifenthrin resistance in hop TSSM populations. Further investigations are essential for deciphering the precise mechanisms through which these genes contribute to acaricide resistance in hop TSSM populations. A comprehensive understanding of their roles could offer novel insights for the development of resistance management strategies and the design of targeted interventions aimed at mitigating acaricide resistance development in TSSM populations from hop fields. The development and successful implementation of the molecular diagnostic tools can further support IPM approach in TSSMs by reducing reliance on the intensive use of acaricides. Additionally, future studies should investigate the role of the formulation components of acaricides used on the genetic architecture of pesticide resistance.

## 4. Materials and Methods

### 4.1. Acaricides and T. urticae Populations

Formulated acaricides were used in the phenotyping of *T. urticae* populations for this study, which included abamectin (Epimek®, 2 EC, now known as Agri-Mek® SC [Syngenta, Hannibal, NY, USA]) and bifenthrin (Bifenture®, 25.1 EC [Solutions Pest & Lawn, Pasadena, TX, USA]). The reference acaricide-susceptible population was collected from a hopyard in Prosser, WA, in summer 2015 [2,23]. Initial characterization in the lab showed that this population has no significant resistance to abamectin and bifenthrin [52]; thus, we subjected the mites to a selection regime to generate resistance. For this, the reference susceptible population was raised on lima beans (*Phaseolus vulgaris* L.) under standard conditions (25 ± 2 °C, 70 ± 5% relative humidity and a photoperiod of 16:8 (light:dark)). A cohort of mites (~500 individuals) was transferred to new lima bean plants and subjected to biweekly selection pressure with gradually increasing doses of commercially formulated abamectin or bifenthrin as outlined in our previous studies [2,52]. This approach ensures a similar genetic background between the susceptible and resistant populations. Selection over 40 generations resulted in an abamectin-resistant population (ABA_1X) at 1× the field dose of abamectin and a bifenthrin-resistant population (BIF_1X) at 1× field dose of bifenthrin, reflecting real-world conditions encountered in hop fields. Selection over approximately 80 generations generated a bifenthrin-resistant population (BIF_100X) at 100× the field dose of bifenthrin, simulating extreme selective pressure. This approach is critical for understanding the escalation of resistance and identifying potential markers associated with high-level resistance. The selection regime and mite maintenance have been described previously [2,23]. Each acaricide-resistant population was maintained in three independent cages to prevent cross-contamination.

### 4.2. Phenotyping Sensitivity to Abamectin and Bifenthrin in the Susceptible and Resistance TSSM Populations

To confirm that repeated exposure to acaricide selection resulted in significant resistance in the respective TSSM populations, adult female mites from each population described above (i.e., SUS, ABA_1X, BIF_1X, and BIF_100X) were subjected to a dose–response bioassay as described previously and reported [52]. Freshly hatched adult females (10–16) were transferred with the aid of a sterile fine paint brush onto a freshly cut lima bean leaf disk (3 cm in diameter). The lima bean disk was placed on water-soaked cotton in Petri dishes and then sprayed with either water or the acaricides using a Potter precision spray tower (Burkard Manufacturing, Richmansworth, Herts, UK) as described in our previous studies [2,23]. There were 3 to 4 independent biological replicates of each acaricide dose tested. There were at least 7 tested doses of each acaricide with the field-labeled rate included. 

### 4.3. RNA Extraction, Sequencing, and Differential Gene Expression

After detecting significant variation in phenotypic resistance to abamectin and bifenthrin, a cohort of ~500 adult female mites was collected from each cage as a biological replication for the SUS, ABA_1X, BIF_1X, and BIF_100X populations, respectively. Each population had three biological replicates. Total RNA was extracted for each replicate in Trizol reagent (Invitrogen, Waltham, MA, USA) using the manufacturer’s protocol. The quality and quantity of RNA were examined using a Nanodrop spectrophotometer (ThermoFisher Scientific, Waltham, MA, USA) and stored at −80 °C. The integrity of the RNA was further examined using the Agilent 2100 Bioanalyzer (Agilent, Santa Clara, CA, USA) at BGI (Beijing Genomics Institute, Beijing, China). Turbo DNase (ThermoFisher Scientific, Waltham, MA, USA) was used to purify the RNA further to eliminate any gDNA, after which magnetic beads were used to isolate poly(A) mRNA. The mRNA was then fragmented and used as a template for cDNA synthesis with random hexamer primers. The cDNA was used in library construction to generate 100 bp paired-end reads. The paired-end sequencing (100 bp) was performed using an Illumina sequencing platform. Deep RNA sequencing was performed using the BGI-SEQ 500 platform (Beijing Genomics Institute, China).

Illumina adapters were trimmed from raw reads and checked for quality using FastQC v0.11.9 (http://www.bioinformatics.babraham.ac.uk/projects/fastqc/, accessed on 22 October 2024). The reads were trimmed and filtered using Trimgalore v0.6.5 (https://www.bioinformatics.babraham.ac.uk/projects/trim_galore/, accessed on 22 October 2024) using the following criteria: minimum read length = 36, minimum read quality = 30. After trimming and filtering, processed pair-end reads were aligned against the scaffold-level TSSM reference genome obtained from Online Resource for Community Annotation of Eukaryotes (ORCAE) database [8] (available at https://bioinformatics.psb.ugent.be/orcae/overview/Tetur, accessed on 22 October 2024) using STAR v2.7.10a [119], keeping all parameters at their default settings. The number of reads aligned to each gene (read counts) was obtained using HTSeq v0.13.5 [120] from an indexed alignment file and supplied gene annotation file in gene transfer format (GTF). The raw read counts were then used to perform differential gene expression in R package Deseq2 [121], which normalized gene expression data based on the median of the ratio method to account for sequencing depth and library composition. Differential gene expressions were measured using the Wald contrast method, statistically comparing the gene expression level between each acaricide-resistant population (ABA_1X, BIF_1X, or BIF_100X) and the susceptible population. The genes were considered differentially expressed if the log_2_fold change was equal to or greater than 1.5 (|log_2_fold| ≥ 1.5) and statistically significant (a Benjamin–Hochberg-adjusted contrast *p*-value of ≤ 0.05).

### 4.4. Gene Ontology Enrichment Analysis

To understand the biological process, molecular function, and cellular components attributed to the differentially expressed genes, gene ontology enrichment analysis was carried out separately for both up- and downregulated genes in all three acaricide-resistant populations using R package topGO v2.54.0 [122]. Gene ontology (GO) terms were considered statistically significant when the Fisher’s exact test *p*-value was < 0.05. The up- and downregulated genes in each enriched GO category were further investigated to understand the potential molecular mechanisms of TSSM resistance to two different acaricides.

### 4.5. Single-Nucleotide Polymorphism (SNP) Calling and Variant Annotation

In addition to differential gene expression analysis, we utilized the RNA sequencing reads to identify functionally important genetic mutations associated with acaricide resistance in TSSMs. The processed high-quality RNA-seq reads were mapped against the TSSM reference genome using a STAR aligner. We used STAR’s two-pass mode to achieve better alignment around novel splice junctions based on the recommendation provided in the GATK method of short-variant identification from RNA-seq data. Sequence alignment files in binary alignment map (BAM) format for each biological replicate of the susceptible or acaricide-resistant sample (ABA_1X, BIF_1X, or BIF_100X) were concatenated, and SNPs were called using the ‘HaplotypeCaller’ function implemented in GATK v4.4.0.0 [123] with all parameters at default settings. The raw variant call file (VCF) was then hard-filtered using the following criteria (QD < 5.0, FS > 60.0, MQ < 40.0, SOR > 4, MQRankSum < −12.5, and ReadPosRankSum < −8.0). Three separate VCF files were generated, each containing genome-wide SNP information for acaricide-susceptible and one of three acaricide-resistant TSSM populations (ABA_1X, BIF_1X, or BIF_100X). Additional filtering was carried out for each file using VCFtools v0.1.16 [124], keeping only biallelic SNPs (--max-allele 2 –min-allele 2), a minor allele frequency of 0.25 (--maf 0.25), a minimum read depth of 10 (--minDP 10), and the maximum missing genotypes per site at 0%. Three separate VCF files were generated, each containing a susceptible sample and one of the three acaricide-resistant samples (ABA_1X, BIF_1X, or BIF_100X). Genotypes in acaricide-susceptible samples were considered as references, and sites were considered as SNPs if the acaricide-susceptible genotype is in a homozygous state and the acaricide-resistant genotype was in either a heterozygous or homozygous state for an alternative allele (Figure S3). SNPs identified in each acaricide-resistant population were annotated using the Ensembl Variant Effect Predictor (VEP) tool [125]. Genes harboring missense mutations in their coding regions were further extracted and investigated for their gene ontology.

## Figures and Tables

**Table 1:** Alignment statistics of RNA-Seq reads in acaricide-susceptible and -resistant two-spotted spider mite (TSSM) samples. (SUS: susceptible; ABA_1X: abamectin-resistant; BIF_1X and BIF_100X: bifenthrin-resistant; R1, R2, R3: first, second, and third biological replicate).

Sample	Biological Replicate	Number of Raw Read Pairs	Number of Filtered Read Pairs	Number of Uniquely Mapped Reads	Uniquely Mapped Reads (%)
SUS	R1	26,987,684	26,978,664	22,341,744	82.81%
SUS	R2	26,576,947	26,566,757	23,258,180	87.55%
SUS	R3	26,827,105	26,818,001	22,323,023	83.24%
ABA_1X	R1	18,504,879	18,497,417	16,229,378	87.74%
ABA_1X	R2	26,623,737	26,613,977	23,281,982	87.48%
ABA_1X	R3	26,883,225	26,874,636	23,320,238	86.77%
BIF_1X	R1	26,767,963	26,760,273	21,344,223	79.76%
BIF_1X	R2	23,505,658	23,499,227	18,466,190	78.58%
BIF_1X	R3	26,661,645	26,653,710	21,460,062	80.51%
BIF_100X	R1	20,019,936	20,013,113	16,857,274	84.23%
BIF_100X	R2	22,661,494	22,653,194	19,333,436	85.35%
BIF_100X	R3	26,754,030	26,742,951	22,731,692	85.00%
Average		24,897,858.58	24,889,326.67	20,912,285.17	84.09%

**Table 2:** Number of up- and downregulated genes in abamectin- and bifenthrin-resistant TSSM populations. Differentially expressed genes for each of the acaricide-resistant TSSM populations were obtained by comparing gene expression with acaricide-susceptible samples. SUS: susceptible; ABA_1X: abamectin-resistant; BIF_1X and BIF_100X: bifenthrin-resistant.

Comparison	Number of Upregulated Genes (log_2_fold Change ≥ 1.5)	Number of Downregulated Genes (log_2_fold Change ≤ −1.5)	Total Number of Differentially Expressed Genes
ABA_1X vs. SUS	28	569	597
BIF_1X vs. SUS	166	469	635
BIF_100X vs. SUS	139	540	679

## Data Availability

Data is contained within the article or supplementary material.

## Supplementary Materials

The following supporting information can be downloaded at: https://www.mdpi.com/article/10.3390/ijms252413298/s1.
